# Snail-Related Contributions from the Schistosomiasis Consortium for Operational Research and Evaluation Program Including Xenomonitoring, Focal Mollusciciding, Biological Control, and Modeling

**DOI:** 10.4269/ajtmh.19-0831

**Published:** 2020-05-12

**Authors:** Fiona Allan, Shaali M. Ame, Yves-Nathan T. Tian-Bi, Bruce V. Hofkin, Bonnie L. Webster, Nana R. Diakité, Eliezer K. N’Goran, Fatma Kabole, Iddi S. Khamis, Anouk N. Gouvras, Aidan M. Emery, Tom Pennance, Muriel Rabone, Safari Kinung’hi, Amina Amadou Hamidou, Gerald M. Mkoji, John P. McLaughlin, Armand M. Kuris, Eric S. Loker, Stefanie Knopp, David Rollinson

**Affiliations:** 1 Wolfson Wellcome Biomedical Laboratories, Department of Life Sciences, Natural History Museum, London, United Kingdom;; 2 Public Health Laboratory - Ivo de Carneri, Pemba, United Republic of Tanzania;; 3 Unité de Formation et de Recherche Biosciences, Université Félix Houphouët-Boigny, Abidjan, Côte d’Ivoire;; 4 Centre Suisse de Recherches Scientifiques en Côte d’Ivoire, Abidjan, Côte d’Ivoire;; 5 Department of Biology, University of New Mexico, Albuquerque, New Mexico;; 6 Neglected Tropical Disease Unit, Unguja, Ministry of Health, Zanzibar, United Republic of Tanzania;; 7 School of Biosciences, Cardiff University, Cardiff, United Kingdom;; 8 National Institute of Medical Research (NIMR) Mwanza Centre, Mwanza, United Republic of Tanzania;; 9 Réseau International Schistosomoses, Environnement, Aménagement et Lutte (RISEAL-Niger), Niamey, Niger;; 10 Center for Biotechnology Research and Development, Kenya Medical Research Institute (KEMRI), Nairobi, Kenya;; 11 Department of Ecology, Evolution and Marine Biology and Marine Science Institute, University of California, Santa Barbara, California;; 12 Swiss Tropical and Public Health Institute, Basel, Switzerland;; 13 University of Basel, Basel, Switzerland

## Abstract

The Schistosomiasis Consortium for Operational Research and Evaluation (SCORE) was created in 2008 to answer questions of importance to program managers working to reduce the burden of schistosomiasis in Africa. In the past, intermediate host snail monitoring and control was an important part of integrated schistosomiasis control. However, in Africa, efforts to control snails have declined dramatically over the last 30 years. A resurgence of interest in the control of snails has been prompted by the realization, backed by a World Health Assembly resolution (WHA65.21), that mass drug administration alone may be insufficient to achieve schistosomiasis elimination. SCORE has supported work on snail identification and mapping and investigated how xenomonitoring techniques can aid in the identification of infected snails and thereby identify potential transmission areas. Focal mollusciciding with niclosamide was undertaken in Zanzibar and Côte d’Ivoire as a part of elimination studies. Two studies involving biological control of snails were conducted: one explored the association of freshwater riverine prawns and snail hosts in Côte d’Ivoire and the other assessed the current distribution of *Procambarus clarkii*, the invasive Louisiana red swamp crayfish, in Kenya and its association with snail hosts and schistosomiasis transmission. SCORE also supported modeling studies on the importance of snail control in achieving elimination and a meta-analysis of the impact of molluscicide-based snail control programs on human schistosomiasis prevalence and incidence. SCORE’s snail control studies contributed to increased investment in building capacity, and specimens collected during SCORE research deposited in the Schistosomiasis Collections at the Natural History Museum (SCAN) will provide a valuable resource for the years to come.

## INTRODUCTION

The Schistosomiasis Consortium for Operational Research and Evaluation (SCORE) was created in 2008 to answer questions of importance to program managers working to reduce the burden of schistosomiasis in Africa. Research related to snails and snail control has been an important part of the SCORE portfolio. This article explores some of the developments and findings relating to wide-scale implementation of snail control and SCORE’s contributions to data and infrastructure that will contribute to future efforts to control and eliminate schistosomiasis.

SCORE studies were primarily designed to:

1) explore the impact of focal mollusciciding in different African settings,
2) investigate methods to detect schistosomes within the intermediate snail host and how these could be used in surveillance of schistosomiasis transmission, and
3) consider the possibility and impact of biocontrol of snail populations within different habitats.

### Historical importance of snail control.

Before the introduction and widespread use of praziquantel and the development of preventive chemotherapy (PC) programs, snail control was considered to be an essential intervention, central to many large-scale schistosomiasis control programs.^1–4^ It continues to be critical in China, where combating the amphibious snail host *Oncomelania hupensis* is essential to the national control strategy.^5^ However, snail control has almost disappeared from the repertoire of programs across Africa, where the availability of praziquantel and the focus on reduction of morbidity has made the PC approach by mass drug administration (MDA) the main, and often the only, intervention being instigated to control schistosomiasis.

Prompted by the World Health Assembly resolution WHA65.21^6^ in 2012, which affirmed the feasibility of eliminating schistosomiasis from some member states, attention is returning to interventions complementary to PC. These will aid reduction of transmission, including strengthened health systems, provision of clean water and sanitation, hygiene education, and snail control.^7^ Indeed, recent models suggest that combining snail control with MDA is a cost-effective strategy for targeting schistosomiasis.^8^

In addition, SCORE carried out a Rapid Answer Project (RAP4)^9^ that addressed: How effective is chemical mollusciciding in reducing snail numbers and in reducing local *Schistosoma* infection risk? This review resulted in three products. The first was a meta-analysis of 63 studies performed between 1953 and 1981, which surveyed a wide variety of approaches to focal snail control in *Schistosoma*-endemic areas.^10^

The major findings from this first study included the following:

1. Snail control reduced schistosomiasis prevalence and intensity in most, but not all locations.
2. The impact of snail control was greater if snail control was combined with MDA.
3. The longer the duration of snail control, the lower were the odds of local human infection.

An outgrowth of this systematic review was the development of two separate historical reviews on the use of snail control for schistosomiasis prevention during the twentieth century.^1,11^ These articles highlighted the fact that well-informed snail control can be an effective schistosomiasis control measure and also serve an important complement to MDA in augmenting program impact.

### Importance of asexual reproduction in snails for the spread of disease.

More than 200 million people who have been infected with schistosomes^12^ share a common experience: they have all been in an aquatic habitat that brought them in contact with cercariae, the infective larval schistosomes that emerge from particular species of freshwater snails. The snail is the essential link that allows the schistosome parasite to develop, multiply, and infect new definitive mammalian hosts via water contact.

Parasite population increase by asexual reproduction (clonal expansion) in the snail hosts. A single miracidium can infect a snail and generate hundreds, even thousands of cercariae, which are released into the water to infect the next definitive host. Therefore, transmission from snails to people can continue in settings where PC has substantially reduced egg output by the human population. Thus, control of snails, or of the schistosome larval stages within them, has the potential for stopping transmission from snails to people.^13^

## CURRENT METHODS FOR IDENTIFICATION OF SNAILS AND INFECTION STATUS WITH SCHISTOSOMES

### Main snail species in Africa and their identification.

In Africa and adjacent regions, two snail genera, *Biomphalaria* and *Bulinus*, are responsible for transmission of *Schistosoma mansoni* and *Schistosoma haematobium*, respectively.^14^ Within each snail genus, different species have their own geographical distributions and compatibilities with different strains and species of *Schistosoma*. As a result, transmission of schistosomiasis varies both within and between endemic areas.^15^ The specificity of the schistosome–snail interaction means that in a particular endemic region, there are typically only one or two species of snails responsible for local transmission. Therefore, it is possible to define places where *Schistosoma* transmission takes place by mapping the occurrence of compatible snails at human–water contact sites (HWCS).

Numerous techniques are available for snail identification. Regional identification keys, such as those produced by the Danish Bilharzia Laboratory, predominantly use shell morphology to identify to family and genus level, although radulae and soft tissue morphology may be needed to delimit further.

Molecular identification methods such as cytochrome c oxidase 1 mitochondrial gene (*Cox1*) sequences are available for numerous freshwater snails (e.g., Refs. 16–18), and reference data are available in online databases such as GenBank and barcode of life data system (BOLD).^19^ As the same *Cox1* region has been sequenced within and between species, this is a good comparison gene for general species identification. However, correct primer selection is critical when dealing with different geographical isolates, in particular for the species of *Bulinus*.^17^ Other genes used for phylogenetic analyses and taxonomic purposes include the ribosomal internal transcribed spacer (ITS), 16S, and 18S.^18,20^ As sequencing technologies progress, the precision of snail identifications can be expected to improve by inclusion of more marker genes, mitochondrial genomes,^21^ or entire genomes.^22^

### Detection of schistosome infections within snails.

Patent infections are defined as snails that are shedding cercariae; such snails contribute to transmission. Snails are generally checked for cercarial emergence immediately after collection or during several days following collection. The emergence of schistosome cercariae peaks at different times, ranging from early morning to late afternoon, depending on the species and target-definitive host. Accurate differentiation among *Schistosoma* species and associated hybrids using cercarial morphology is not routinely feasible; molecular analysis is needed to confirm the species.^23,24^ This is important for evaluating transmission and testing assumptions made in relation to the schistosome species being transmitted, based on geography, the type of study being conducted, and the snail species involved.^25,26^

The prepatent (or latent) infection period spans the time between penetration of the snail by a miracidium and the emergence of cercariae. For *Biomphalaria* and *Bulinus* species, this period may range from 20 to 35 days or longer, depending on ambient temperature and the number of miracidia having penetrated the snail and snail-specific factors such as size, age, health, nutrition, crowding, and most importantly the level of snail–schistosome compatibility.

Typically, schistosome infections within snails are detected by direct observation (via microscopy). Snails are crushed or dissected and examined for the presence of sporocysts, or they are allowed to shed cercariae, generally either at one time point or over an extended period.^27^ More recently, scalable, flexible, and potentially high-throughput techniques are being used. With a few exceptions, such as use of monoclonal antibodies/ELISA,^28^ these methods involve amplification of schistosome DNA within the snail host.

Currently, the most commonly used approach to detect infections in snails involves amplification of DraI (*S. haematobium* group) or SM1-7 (*S. mansoni* group), which are repetitive genetic elements.^29–31^ Specificity may be problematic with regard to the discrimination among species of the *S. haematobium* group, particularly using DraI in mixed infection areas, although additional refinements have been made to unambiguously identify species.^32^ In any given field situation, it is necessary to validate the results produced by these methods by amplification and sequencing of species-specific amplicons. Methods of detection have moved in some cases from polymerase chain reaction (PCR) amplification to loop-mediated isothermal amplification (LAMP). This has the advantage of reducing the amount of expensive equipment needed to perform analyses in the field.^33,34^

Progress in moving from proof of principle studies, using several different methods, to large-scale field assessment has been slow. However, studies have shown that in Kenya, prepatent infection prevalence far exceeds the prevalence of snails shedding *Schistosoma* cercariae.^31^ Similarly, DraI was used to measure prepatent infections in *Bulinus globosus* at several field sites in Zanzibar from 2003 to 2007.^35^ Again, the number of snails harboring prepatent *S. haematobium* infections was considerably higher than the number of snails shedding cercariae. The recent finding of sympatric *Schistosoma bovis* in the north of Pemba^26^ emphasizes the need for awareness of the specificity of the target gene.

More recently, a sensitive mitochondrial marker for the detection of *S. mansoni* infections of *Biomphalaria sudanica* and *Biomphalaria pfeifferi* in Kenya was developed by Lu et al.^25^ This used end point PCR to amplify and visualize (by agarose gel electrophoresis) a small region of the mitochondrial ND5 gene. The resulting specifically sized PCR amplicon for *S. mansoni* provides a one-step diagnostic PCR. Although the PCR also amplifies *Schistosoma rodhaini* (the sister species to *S. mansoni*), the amplicon produced is larger than that for *S. mansoni*. However, confirmatory sequencing should be carried out, when possible, to clarify which of these two species is present. This assay was also tested on field samples, proving the assay’s ability to identify prepatent infections.

## SCORE PROJECTS RELATED TO SNAILS

The SCORE agenda was shaped with the input of experts from many different backgrounds in addition to those whose work is focused on schistosomiasis.^36^

Inclusion of snail-related studies within SCORE was considered a high priority. Therefore, SCORE has supported work on snail identification and mapping, and how xenomonitoring, the detection of pathogen infections within the vector, can assist in the identification of infected snails and thereby identify and characterize potential transmission foci. Moreover, focal mollusciciding, with niclosamide, was undertaken both in Zanzibar and Côte d’Ivoire as part of elimination studies. In addition, two studies involving biological control of snails were conducted: one explored the association of freshwater riverine prawns and snail hosts in Côte d’Ivoire and the other assessed the current distribution of the invasive *Procambarus clarkii*, the Louisiana red swamp crayfish, in Kenya and its association with intermediate snail hosts and schistosomiasis transmission. SCORE also supported modeling studies on the importance of snail control in achieving elimination and a meta-analysis of the impact of molluscicide-based snail control programs on human schistosomiasis prevalence and incidence. SCORE’s snail control studies contributed to increased investment in building capacity, and the specimens collected during SCORE research, stored in Schistosomiasis Collections at the Natural History Museum (SCAN), will provide a valuable resource for years to come.^37^

### Field studies.

SCORE’s largest field studies compared alternative approaches to gaining and sustaining control of schistosomiasis (the gaining and sustaining control studies). These studies are described in detail in Ref. 38. As part of these studies, freshwater snail distributions and schistosome infections in the Niger River Valley^39^ and on the shores of Lake Victoria in Tanzania^40^ and Kenya were evaluated, and cercariae from some locations were characterized, as described in Ref. 41. At the end of the gaining control study, two further projects were carried out; one in Kenya to examine the differences in snail infection levels between villages where the prevalence and intensity of infection among children did and did not decline after 4 years of MDA; and second in Mwanza, Tanzania, to evaluate the snail host distributions and the levels of patent snail infections in villages characterized as transmission persistent hot spots (PHSs) and responders after 4 years of MDA. In the Tanzanian study, molecular xenomonitoring of nonpatent infections was used to evaluate if this method gives a more sensitive measure of transmission in places with low-level *S. mansoni* transmission. Two multiyear intervention studies—one in Zanzibar^42,43^ and one in Côte d’Ivoire^44^—evaluated the impact of adding focal mollusciding with niclosamide to MDA on human prevalence and intensity of schistosomiasis. Two observational studies were conducted—one in Côte d’Ivoire^45^ and one in Kenya^46^—to explore the possible relationships between crustaceans, snail populations, and human infection.

#### Snail monitoring in the Tanzania gaining control and Niger studies.

The SCORE studies of gaining control of schistosomiasis were conducted over 4 years of intervention, with 150 villages in each country randomized to receive MDA using different approaches (school-based or community-wide) and on different schedules (annual, biannual, and biennial). Countries originally involved in gaining control studies were Kenya, Mozambique, Niger, and Tanzania.^38^ In Year 3, because of inadequate randomization of villages, the Niger study was redesigned to involve twice-a-year versus once-a-year treatment. Snail and schistosome population genetics studies were embedded in the gaining control study in Tanzania and the study in Niger.

Human prevalence and intensity of schistosomiasis were measured before the start of the study, before each MDA, and at the study end. Snails, as well as cercariae and miracidia for population genetics testing, were collected from selected villages in the study arms in Tanzania and Niger receiving the most intensive MDA and an arm that received less-intensive treatment.

In Tanzania, 42,874 *Biomphalaria* were collected across 26 sites and over 4 years, of which 509 (1.2%) were shedding schistosome cercariae. Local knowledge of water contact behavior was used to choose the study sites. *Biomphalaria sudanica* is the main *Biomphalaria* species involved in transmission in the study area, but there were significant site variations, with *Biomphalaria choanomphala* strongly involved at some sites. The highest number of shedding *Biomphalaria* was found at sites with high human movement, for example, major fishing/commuting/trading sites. There was no significant difference in the numbers of shedding *Biomphalaria* between seasons, but the number of shedding snails was significantly lower in 2015 (Year 4) relative to 2012 (Year 1). Abundance of *B. sudanica* displayed seasonality, with the highest number of snails collected in the dry season. In a setting like Lake Victoria, it would be more practical to apply snail control in the marshes and small water bodies on the banks of the lake during the dry season when snail numbers/density are higher than those during the rainy season when snail numbers are lower and/or snails are more dispersed. However, snail control may not be a feasible approach to reduce transmission at sites where *B. choanomphala* is implicated in transmission, as this is a deeper dwelling species. Targeting high-risk areas (fish markets and commuting ferry ports) using integrated, combined approaches, such as MDA, snail control, and WASH, may be needed to control transmission^40^.

In Niger, 68 potential transmission sites were identified in the 16 SCORE villages selected for snail and population genetics studies. Snail surveys were conducted monthly from July 2011 to January 2016 at these sites and others from a previous study^39^ (Pennance et al., in preparation) During this period, 87 shedding bulinid snails were found at the SCORE sites. Using microsatellites, multiple schistosome infections per snail were identified. Barcoding cercarial genotypes (*n* = 186) revealed four species types: *S. haematobium*, *S. bovis*, *S. haematobium–bovis* hybrids, and *S. haematobium-bovis-curassoni* hybrids. The distribution of the snails shedding the human-infecting schistosomes (*S. haematobium* and the *S. haematobium* hybrids) strongly correlated with the prevalence of urogenital schistosomiasis in the local communities. However, the finding of hybrids raises the question of whether nonhuman reservoirs may contribute to ongoing transmission^39^. The detailed mapping of potential transmission sites and the species being transmitted would be potentially very useful for targeted snail control interventions, habitat modification, and interventions to limit human contact at sites with infected snails.

#### Xenomonitoring in low-endemic areas.

The aim of a SCORE study in low-endemic villages in the Mwanza region, Tanzania, was to determine if xenomonitoring could be used as a sensitive tool for detecting *S. mansoni* transmission where prevalence had reached low levels after continued MDA. Here, traditional snail collecting and cercarial shedding (with molecular identification of the cercariae) was coupled with molecular prepatent screening of *Biomphalaria* snails collected from three low-prevalence *S. mansoni* villages, on the shores of Lake Victoria, where treatment interventions had reduced the prevalence of human infections (6–11%) by 78% or more over four rounds of annual MDA (2012–2016). As a comparison, snails were also surveyed and examined for patent *Schistosoma* infections (cercarial shedding) from three villages classified as being transmission PHSs (prevalence is persistent despite several rounds of MDA and did not reduce by more than 35% over four rounds of annual MDA), but only snails from one of these villages were further analyzed for prepatent infections. Snails were collected at four time points (every 3 months over a year) to assess whether there were seasonal differences in snail infection rates. For prepatent screening, the diagnostic ND5 assay^25^ was optimized to include an internal control (snail DNA amplicon ITS^17^) to check for false negatives in relation to failed DNA extractions or PCR inhibition. All PCR amplicons were sequenced to confirm schistosome species, as it was observed that *S. rodhaini* was as prevalent in these areas as *S. mansoni* and could not always be distinguished by amplicon size.^25^

More than 10,000 snails from 35 HWCSs, places where human–water contact was taking place, for any activity including, bathing, household chores, and fishing. All sites were identified with the assistance of a village leader. The three low-transmission villages were targeted and the snails were collected and screened for infection. Snails were collected, semiquantitatively, by two people for a period of 15 minutes at each site; vegetation and potential snail habitats within the vicinity of the HWCSs were surveyed. Snails shedding *S. mansoni* cercariae were found in all villages (overall prevalence of 0.1%), in at least one of the time points with no relation to season. Thus, in these low-prevalence sites, this method was sensitive enough to identify low levels of ongoing transmission. However, snail distributions were focal; shedding snails were only found at certain sites, which suggests focused transmission. Molecular prepatent screening of individual snails identified around 8% more *S. mansoni* infections than patent infections. Higher levels of prepatent infections in comparison with patent infections (≳ 51%) have been found in previous studies, but these data were from the *S. haematobium–Bulinus* system.^35^ Shedding snails were found in the PHSs at a similar level to the villages that had low prevalence in the human population; this was also true of prepatent snail infections detected in the one PHS village included in the molecular screening.

This study demonstrated that large-scale snail xenomonitoring could detect transmission in low-endemic settings, with prepatent screening resulting in increased prevalence of infections (patent and prepatent). In this study, the numbers of infected snails found in PHS versus low-transmission areas were not considerably different highlighting the usual observations of low levels of infected snails found in many transmission-monitoring studies, independent of the level of transmission. The findings also highlight the need for species-specific markers, particularly as animal schistosome species are often abundant in these types of setting. The absence of infection in snails can also be informative. This was observed in Bulungwa village where transmission was assumed to be persisting at a very low level (∼1%); however, this village was far from the lake shore and it was not known where transmission was taking place. Surveys were carried out at different time points, but no suitable water bodies were found that either contained *Biomphalaria* snails or where there was HWCS. This suggested that these few cases of infection were actually imported cases and there was no local transmission and indicated further work may be required to ascertain if water contact is occurring at this village.

It does have to be noted that the surveying, collection, and snail screening methods can be laborious and must be performed accurately. Large numbers of snails need to be collected, processed quickly, and analyzed efficiently. However, the methods are inexpensive, sustainable, and are potentially transferable to be performed in countries enabling the local teams to run their own xenomonitoring studies independently, thus increasing their capacity for monitoring transmission and control.

#### Snail monitoring in persistent hot spot and responding villages in Kenya.

One result of the SCORE gaining control studies was the finding that annual administration of praziquantel (PZQ) had differing effects among different villages. At the end of the Kenya gaining control study, an additional effort was made to see if factors could be identified that were associated with a village being a PHS (≤ 30% reduction in prevalence and/or ≤ 50% intensity reduction following repeated treatments) or a responder village (> 30% prevalence and/or intensity reduction following four years of MDA). Possible inherent differences in snail-related aspects of transmission were investigated in 10 of these villages, six of which were identified as PHS villages, and four of which were responders, locations of which along with survey times and methods are detailed in the associated publication.^46^ Initial prevalence values were 60.5–91.8% for PHS villages and 52.9–81.6% for responder villages.^46^ Villages that were PHSs were located along the west-facing shore of the lake and responders faced the Winam Gulf, suggesting the potential importance of ecological factors*. Biomphalaria sudanica* did not differ in the relative abundance or prevalence of *S. mansoni* infection between PHSs and responding villages, when all sampling sites, times, and water hyacinth presence/absence were taken into account. *Biomphalaria choanomphala*, a snail species typically found in deeper water, was significantly more abundant in the PHS villages. Furthermore, the abundance of *B. choanomphala* among villages was positively correlated with *S. mansoni* prevalence in children in the studied villages. It is possible that an alternative mode of transmission provided by *B. choanomphala* facilitates greater persistence of *S. mansoni* in PHS villages. In addition to assessing snails, sentinel mice were exposed to water in floating cages at sampling sites. Recoveries of *S. mansoni* from sentinel mice occurred but did not differ between PHSs and responder villages.^46^

All 10 villages we examined provided evidence for potential ongoing *S. mansoni* transmission (either because of infected snails or positive sentinel mice), leading to the conclusion that conditions conducive for transmission and reinfection occur throughout the study area. Owing to rapid reinfections facilitated by large snail populations and movements of infected people around the lake, sustained control of schistosomiasis in this area may require an integrated, persistent, basin-wide approach.^46^

### Studies involving snail control with niclosamide in Zanzibar and Côte d’Ivoire.

Niclosamide is currently the molluscicide most widely used for snail control. Because of its broad activity against nontarget organisms and high cost, a targeted approach to control snails in habitats directly associated with transmission—focal application is the preferred approach of use for molluscicides.^47^ Focal mollusciciding with niclosamide aims to minimize environmental impact, also ensuring treatment is only of areas that are contributing to schistosomiasis transmission. Careful application is key, as detailed in the WHO Operational Manual for Program Managers (2017).^48^ Community sensitization and robust site assessments are required, and teams carrying out mollusciciding must be fully trained in methods for focal spraying and wear essential protective gear.

The SCORE studies in Zanzibar and Côte d’Ivoire included intervention arms that combined MDA and snail control. In both of these cluster-randomized trials, randomly selected villages received MDA + snail control. Human *S. haematobium* infection prevalence and intensity at the end of the study within the MDA + snail control arm were compared with those of people in villages randomized to other interventions.

The approaches to niclosamide use in Zanzibar and Côte d’Ivoire were similar in many ways. In both studies, a critical early step was to seek local support for use of niclosamide and identify water bodies with human–water contact where niclosamide might potentially be applied. Community leaders were helpful in both of these aspects. In Zanzibar, the reasons for snail control in the schistosomiasis elimination program were discussed in community meetings and with local leaders. The success of these discussions resulted in requests for mollusciciding from communities that were not in the snail control arm, which could not be provided during the SCORE project as it would have invalidated the study. In Côte d’Ivoire, the study team met with village leaders when surveys were being conducted to determine the eligibility of villages for the study. Engagement with communities was highly participatory. For example, in one Côte d’Ivoire region, the snail team consulted with the youth committee for accurate identification of water bodies and HWCSs.

Protocols for field surveys and field application of niclosamide were similar in both studies. At each visit to an HWCS, the type of water body, GPS coordinates, and physical and environmental features of the site were recorded. Snails were collected according to protocols that specified the amount of time for collection, distance along the shore that would be assessed, depth of collection, and other parameters. Details of snail species identified were recorded. In the field, niclosamide was only applied to water bodies where there was human contact and intermediate host snails were present.

#### Zanzibar elimination study.

Zanzibar has long been recognized as an endemic area for urogenital schistosomiasis. However, the control of this disease was not comprehensively implemented until the late 1980s, when selective chemotherapy or school-based treatment with praziquantel began. This control effort resulted in a dramatic reduction in macroscopic hematuria and parasite prevalence. In 2004, Zanzibar received financial and technical support from the Schistosomiasis Control Initiative to implement a multiyear project to control the morbidity of schistosomiasis through MDA. In 2011, the Government of Zanzibar and partners created the Zanzibar Elimination of Schistosomiasis Transmission (ZEST) project to investigate multidisciplinary control measures including snail control.^43^ A SCORE-supported “Zanzibar elimination study” of alternative approaches to elimination of schistosomiasis was embedded within ZEST.^49^

The Zanzibar elimination study was conducted on Unguja and Pemba islands—the major islands of Zanzibar. Forty-five shehias (the smallest administrative unit in Zanzibar) per island were randomized to one of three arms: biannual MDA, biannual MDA + snail control, and biannual MDA + behavioral interventions.^43^

In the study arm with snail control, snail assessments and niclosamide treatment were conducted during 8 months per year (from August to March) during the entire project period (from mid-2012 until early 2017). The time periods for assessment and intervention were chosen so as to avoid periods of heavy rains (April–June).

**Figure 1.**
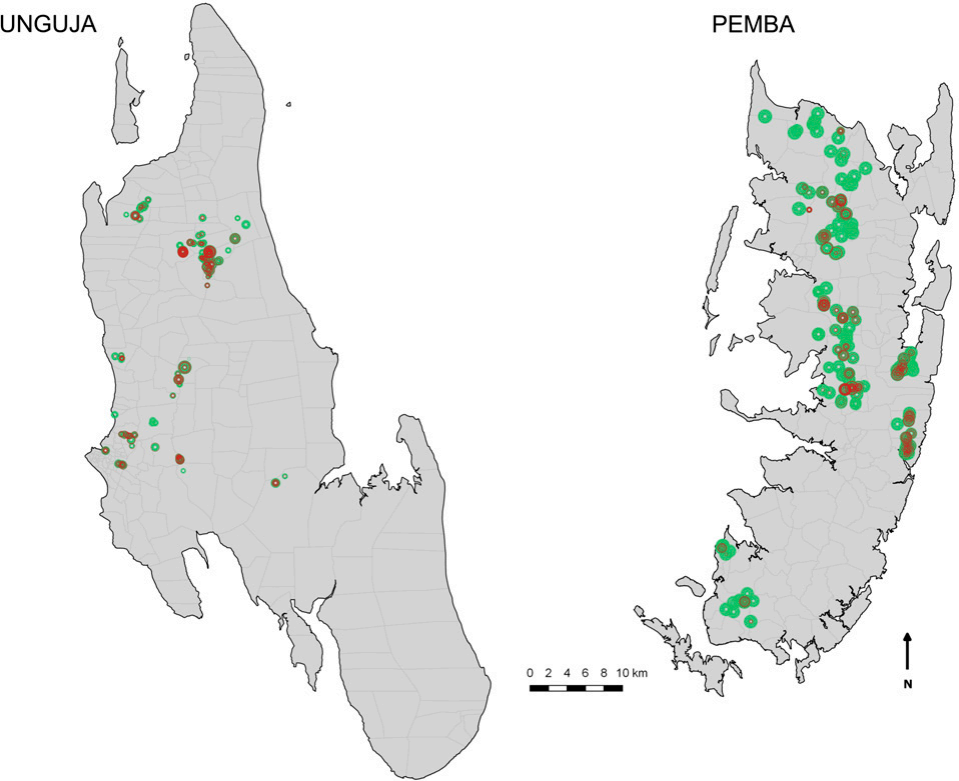
Maps showing the human–water contact sites in the 15 shehias on Unguja and Pemba, respectively, that were randomized to snail control in the Zanzibar elimination study. Dot size represents the number of surveys (1–23) conducted between 2012 and 2016 and whether *Bulinus* spp. were present (red) during one or several of the surveys.

A large number of water bodies were identified on both islands. The number increased over time because of increased assistance from local communities in finding them and potentially also heavy rains resulting in new temporary water bodies. A total of 167 and 121 HWCSs were surveyed in Pemba and Unguja, respectively. As shown in Table 1, the highest numbers of *Bulinus* on both islands were found before snail control interventions started—2,599 *Bulinus* in 45 HWCSs in Pemba and 1,716 *Bulinus* in 29 HWCSs in Unguja. In subsequent years, the number of *Bulinus* collected ranged from 221 to 1,012. However, there was no decreasing trend over the years from 2013 through 2016 and snail populations recovered after spraying (Table 1, Figure 1). The percentage of *Bulinus* shedding schistosome cercariae by visual inspection was low: 0.2% in Pemba and 0.9% in Unguja.

**Table 1:** Human–water contact sites (HWCSs) surveyed, *Bulinus* spp. found, and mollusciciding coverage in Zanzibar, by year and island.

	2012	2013	2014	2015	2016	Total
Pemba						
Number of HWCSs surveyed	140	139	143	143	139	167
Number of HWCSs with *Bulinus* spp. (%)	45 (32.1)	46 (33.1)	45 (31.5)	42 (29.4)	29 (20.9)	71 (42.5)
Number of HWCSs with *Bulinus* spp. treated with niclosamide (%)	38 (84.0)	43 (93.5)	42 (93.3)	41 (97.6)	26 (89.7)	60 (84.5)
Number of *Bulinus* spp. collected	2,599	788	795	1,012	384	5,578
Number of *Bulinus* spp. shedding	4	4	1	0	0	9
Number of days team worked in the field	63	113	153	136	87	552
Unguja						
Number of HWCSs surveyed	39	40	91	105	111	121
Number of HWCSs with *Bulinus* spp. (%)	29 (74.3)	22 (55.0)	47 (51.7)	35 (33.3)	50 (45.0)	91 (75.2)
Number of HWCSs with *Bulinus* spp. treated with niclosamide (%)	9 (31.0)	19 (86.4)	33 (70.2)	29 (82.9)	36 (72.0)	65 (71.4)
Number of *Bulinus* spp. collected	1,716	565	676	221	785	3,963
Number of *Bulinus* spp. shedding	0	13	17	0	5	35
Number of days team worked in the field	29	20	60	96	141	346

Adapted from Knopp et al.^41^

Of HWCSs containing *Bulinus*, 85% in Pemba and 71% in Unguja were treated with niclosamide. As shown in Figure 1, from 2012 through 2016, Pemba conducted up to 23 snail surveys per HWCS and 23% of all HWCSs were sprayed 10 times or more, whereas Unguja conducted up to 20 surveys per HWCS and 16% of HWCSs were sprayed at least five times. A total of 1,600 kg of niclosamide were used during the study.

**Figure 2.**
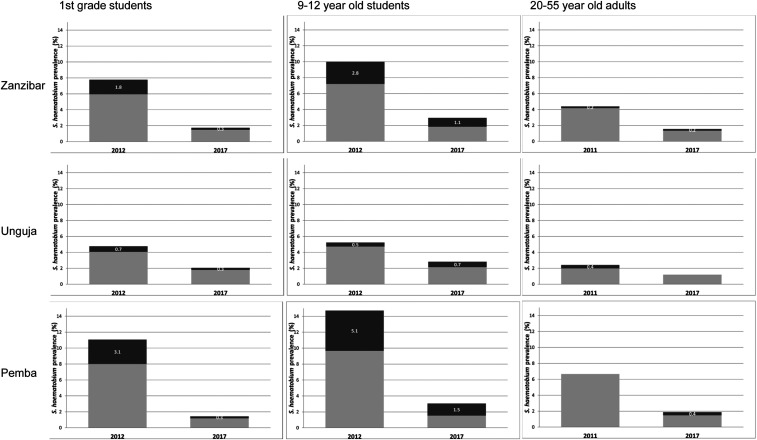
*Schistosoma haematobium* prevalence and intensity in first grade, 9–12-year-old children, and 20–55-year old adults in the snail control arm in Zanzibar (overall and by island), at baseline in 2012 and end line in 2017. Bar charts present the *S. haematobium* prevalence. Dark gray parts with white numbers present the percentage of heavy infection intensity among the overall prevalence.

In the areas where snail control was applied in Pemba, *S. haematobium* human prevalence decreased over the study period from 11.1% to 1.4% in 9- to 12-year-old children, 14.7% to 3.1% in first-year students, and 6.7% to 1.9% in 20- to 55-year-old adults.^42,50^ In the Unguja snail control study arm, the *S. haematobium* prevalence decreased from 4.8% to 2.1% in 9- to 12-year-old children, 5.3% to 2.8% in first-year students, and 2.4% to 1.2% in 20- to 55-year-old adults. The decrease in *S. haematobium* prevalence and infection intensity in the snail control study arm from 2011/12 until 2017 is shown in Figure 2. After adjustment for baseline prevalence, a trend of lower odds of *S. haematobium* infection in 9- to 12-year-old children attending schools in shehias that received biannual MDA + snail control compared with those in biannual MDA-only shehias was observed from 2015 onward.^42^ However, the difference between the arms was not statistically significant. In some years, the *S. haematobium* prevalence appeared to rebound after having declined; this was more often observed in shehias with high baseline prevalence.

The application of niclosamide did on occasion have negative impacts on aquatic animals, especially fish. Surprisingly, the deaths of a few ducks were reported in areas where niclosamide was applied, but it was not established whether these deaths were caused by niclosamide or if other factors were responsible. Nevertheless, community acceptance of mollusciding remained high because of investment in sensitization of all the communities.

#### Côte d’Ivoire seasonal elimination study.

Côte d’Ivoire is endemic for schistosomiasis.^51,52^ Until 2015, the mainstay strategy of schistosomiasis control in Côte d’Ivoire was MDA with praziquantel, to achieve control rather than elimination. In line with the WHO goals and recommendations for eliminating schistosomiasis in endemic countries,^2,53^ a SCORE study of elimination in a seasonal setting began in early 2016, to evaluate alternative approaches to interrupting the transmission of *S. haematobium.* The study was carried out in the northern and central regions of the country, where both *B. globosus* and *Bulinus* *truncatus* are potential intermediate snail hosts for *S*. *haematobium*. In these regions, there is strong seasonality in the presence of snails and schistosomiasis transmission. The study included four arms—three of which involved providing MDA alone on different schedules. The fourth arm combined annual MDA with snail control using niclosamide.^44^

Before this study, mollusciciding for snail control had never been implemented in Côte d’Ivoire. Therefore, approval for exceptional use of the product had to be obtained from the national committee for pesticide use (Comité Pesticides de Côte d’Ivoire, Ministère de l’Agriculture). Because it was new to the country, implementing molluscidicing was challenging in terms of both sensitization of communities and developing the capacity to conduct field and laboratory work.

A parasitological survey was conducted in November 2015 to identify 64 villages that were eligible for the study, with criteria being *S*. *haematobium* prevalence ≥ 4% among 13–14-year-old children. Sixty-five villages were enrolled at first, but one refused access to water bodies and withdrew, leaving 64 villages in the study. The snail team was formed in February 2016 comprising two postdoctoral students, a PhD student, and three technicians. Training on aspects of snail collection and field collection data, niclosamide preparation and application, and cercariae preservation and identification was carried out by members of the Natural History Museum (NHM, London, United Kingdom) team.

After randomization into the four arms, a baseline malacological survey was conducted in areas around the 16 villages in the MDA + snail control arm—half of which were in the northern and half in the central part of Côte d’Ivoire.

Snail surveys were conducted three times per year: before the peak transmission season of schistosomiasis (in November), during the peak transmission season (in February/March), and shortly after the peak transmission (in June). Mass drug administration in the MDA + snail arm was carried out once a year, after the November mollusciciding. All surveys were conducted by the same two investigators. In the laboratory, snails were counted and identified using standard identification keys. Following each of the three annual snail surveys, niclosamide was applied focally to HWCSs with *Bulinus* spp. present.

*Bulinus* spp., specifically *B*. *truncatus*, *B*. *globosus*, and *B. forskalii*, were screened for infection individually by inducing cercarial shedding and observation under a stereomicroscope.^54^ All samples were accessioned into the schistosomiasis collection at the NHM (the SCAN project—see Ref. 37) and a subset were characterized by PCR, as described in Tian-Bi et al.^23^

Five types of water bodies were identified: dammed and natural lakes, irrigation canals, streams, rice paddies, and ponds. A total of 164 HWCSs were surveyed, with the greatest number found at the end of the rainy season (June and November) and lowest numbers found during the dry season (in February/March), many of the HWCSs being seasonal/ephemeral.

The field visits for identifying HWCSs and snail sampling were challenging because of the large size of water bodies to be surveyed/treated and long distances between study localities. Splitting the team into two for later surveys overcame some of these problems.

More than 15 HWCSs harbored snail hosts of schistosomes and more than 4,000 *B*. *truncatus* and *B*. *globosus* were collected, 60 of which shed schistosome cercariae (1.5%). The study found *Bulinus* transmitted not only human schistosomes *S*. *haematobium* and *S. haematobium–bovis* hybrids but also the bovid schistosome *S. bovis* (transmitted by a large proportion. Human schistosomes were transmitted in both the northern and central sites; however, there were geographical trends in the relative distributions of schistosome species with the snails collected from northern sites predominately infected with *S*. *bovis*, whereas those sampled from central sites were mainly transmitting *S*. *haematobium* and *S. haematobium*–*bovis* hybrids.^23^

On the whole, mollusciciding was welcomed by the residents of the chosen villages. Mollusciciding was seen to reduce snail abundance in most cases, and the number of schistosome-infected snails decreased. However, recolonization of snail habitats was observed (mostly at the second visit after treatment). An overall decrease in the number of schistosome-infected snails over time was observed. Within the human population, there was also a decrease in infection prevalence by 3.4% over all the study sites (baseline prevalence of *S. haematobium* in 9- to 12-year-old children was 24.8%).

To monitor the population dynamics of nontarget aquatic organisms over time, an inventory focusing on species and abundances was conducted before the intervention and following the malacological survey in MDA + snail control villages. Six villages from the non-snail control arms were also surveyed to serve as controls. Although nontarget aquatic organisms were sometimes impacted, the impacted organisms recolonized habitats between niclosamide applications.

### SCORE studies involving biological control through predation.

Biological control of snails to reduce schistosomiasis involves using living organisms, such as fish, birds, or crustaceans, to affect the abundance of freshwater snails.^3,56^ Use of natural snail predators for control has the potential to be sustainable, not requiring frequent reapplication as is required with niclosamide.^45^ Recent studies along the Senegal River suggest that the prawn, *Macrobrachium vollenhovenii*, feeds on intermediate host snails and may have a positive impact on schistosomiasis transmission reduction.^3^ Therefore, SCORE supported studies in two countries to contribute to understanding the potential for biological interventions as a strategy for schistosomiasis control.

#### Riverine prawns in Côte d’Ivoire and association with freshwater snail hosts.

SCORE funded a study in Côte d’Ivoire with two specific aims: 1) to determine the relationship (prevalence and density) between native riverine prawns and intermediate host snails and 2) to explore associations between specific native riverine prawns, intermediate host snails of schistosomiasis, and the prevalence of *Schistosoma* infection in humans. The investigation was carried out in two purposely selected coastal river systems in the southeastern part of Côte d’Ivoire.^56^ A single cross-sectional parasitological survey among humans in 24 villages was conducted near the Agnéby and Mé coastal river systems in southeastern Côte d’Ivoire. At each site, snails and prawns were collected, and in each village, 150 individuals participated in the study, contributing stool and urine samples for testing for *S. mansoni* and *S. haematobium*.

Overall, a negative association was observed between intermediate host snail densities and riverine prawns. However, no pattern was found between this trend in the predator–prey relationship and the prevalence of human schistosomiasis. Snails were found to be shedding schistosome cercariae in 10 of the 24 villages surveyed (six villages in Agnéby River system and four in the Mé River systems) in at least one of the four seasons when they were surveyed. Both *B. pfeifferi* and *B. globosus* were found to be infected in eight of the villages.^45^

The total number of participants in the parasitological survey was 3,338 (2,378 children and 960 adults). Stool samples were provided by 3,227 individuals (2,319 children and 908 adults), and *S. mansoni* eggs were found in 381 (11.8%). Urine samples were provided by 3,204 people (2,306 children and 898 adults), and *S. haematobium* eggs were observed in 216 (6.7%). The prevalence of *S. mansoni* infection was significantly higher in the Mé River system than in the Agnéby River system (19.1% versus 5.5%; odds ratio (OR) 4.0, 95% CI 3.1–5.1). By contrast, *S. haematobium* prevalence was higher in the Agnéby River system than in the Mé River system (8.0% versus 5.2%).^45^

In this study, *Macrobrachium* spp. prawns were present in the main river courses that are well oxygenated, whereas the intermediate host snails of schistosomiasis were mainly present in the secondary tributaries. Hence, predatory prawns and intermediate host snails were rarely observed in the same water bodies. Although the presence of intermediate host snails was positively correlated with total dissolved solids, pH, and clear water, predatory prawns showed a positive relationship with redox potential, resistivity, and clear water. The abundance of intermediate host snails and their levels of schistosome infection did not correlate with human infection prevalence.

#### Follow-up assessment of *Procambarus clarkii*

In the late 1980s, several prospective agents for the biological control of schistosome-transmitting snails were investigated in Kenya.^57^ In the course of that work, it was observed that schistosome-transmitting snails rarely, if ever, coexisted in water bodies with invasive *P. clarkii*, the Louisiana red swamp crayfish. *P. clarkii* is a voracious snail predator. It also serves as a competitor, as it readily consumes aquatic plants used by snails for cover and oviposition sites.

The precise details of when and how this introduced crustacean arrived in East Africa remain obscure. It may have been introduced into Kenya for aquaculture purposes in the 1960s,^58,59^ but it is possible it was introduced even earlier in the 1950s.^46^ Following its introduction, *P. clarkii* dispersed widely throughout Kenya. By the early 1990s, it was found in all major drainage basins within the country.^58^

Investigations of *P. clarkii* and its potential ability to control schistosome-transmitting snails showed a strong negative correlation between the presence of *P. clarkii* and schistosome-transmitting snails in Kenyan freshwater habitats. Furthermore, crayfish eradicated or greatly reduced snail populations in both laboratory and field enclosures.^60–62^ In addition, a field trial was initiated in 1989 to test the effects of the presence of *P. clarkii* on transmission of *S. haematobium* in the rural area around Kitui, Kenya.^63^ Six schools were divided into three experimental control pairs. At experimental schools, crayfish were introduced into nearby aquatic habitats with *Bulinus africanus* snails and serving as *S. haematobium* transmission sites. Snail habitats near control schools did not receive crayfish. In one, of three, experimental areas where *P. clarkii* became well established, schistosomiasis levels in children decreased markedly compared with those in the matched control school. This crayfish effect was most evident when transmission sites were not prone to sustained drying. In an unrelated development, *P. clarkii* has since become established in Egypt, where a negative correlation between the presence of crayfish and the prevalence of both *S. mansoni* and *S. haematobium* infection has also been found.^64,65^

Given its potential as a control agent, SCORE funded a follow-up survey to investigate the current status of *P. clarkii* in previously investigated habitats in Kenya, including the vicinity of the primary schools in Kenya where the use of *P. clarkii* to control schistosomiasis had been studied 18 years prior.^63^ This survey consisted of a single visit to 13 habitats in January or March 2014. Snails were surveyed using standard scooping and hand collection methods, and crayfish were collected by scooping or trapped using submerged meat-baited traps made of nylon mesh that were left in situ for 1 hour.

The results of this survey indicated that crayfish have persisted in five of nine of the habitats where they were known to be present over 20 years ago. These include prominent habitats such as Lake Naivasha and dams in Eldoret and Kinyanga. We also noted the presence of crayfish in parts of the large Mwea rice growing scheme where they were absent during the 1980 crayfish studies. A search of the ephemeral habitats associated with the 1992 Kitui-based study^63^ of the effects of crayfish on *S. haematobium* transmission did not yield crayfish nor were *S. haematobium*–transmitting snails recovered from habitats where they used to exist before crayfish introduction (Table 2). One of the two control sites we were able to visit where crayfish were not introduced in 1989 continued to harbor a population of *B. africanus*, a known *S. haematobium* vector.

The survey highlighted that the aquatic habitats sampled are dynamic and subject to change. In some cases, as with our ephemeral Kitui study sites, disappearance of crayfish was associated with draining or drying of habitats, making it difficult to draw any firm conclusion about the current impact of crayfish on *S. haematobium* transmission there. In the Sirikwa Dam in Eldoret, we noted a reduction in crayfish abundance and a reemergence in vector snail populations. The surprising presence of crayfish in the large Mwea scheme bears further scrutiny because this is an important endemic focus for *S. mansoni* transmission. Vector snail species, including *B. pfeifferi* and *Bulinus* sp., are also present there, and current patterns of coexistence with crayfish are unclear and may be influenced by insecticide impacts on crayfish. By contrast, Lake Naivasha has been more stable in persistently supporting a thriving crayfish population for 50 years. The schistosome vector snail *B. sudanica* once reported from the lake was not seen there by us in the 1980s nor during our recent survey. The lake supports large populations of introduced North American gastropods, including *Physa acuta* and *Pseudosuccinea columella*.

### SCORE modeling work on snail control.

After the RAP4 project cataloged the range of effectiveness observed in past snail control programs, the results were used as inputs for predictive modeling to estimate the likely incremental benefits of adding snail control to modern-day MDA programs.^8^ Using the latest stratified worm burden simulation model of community-level schistosome transmission and human prevalence (see Ref. 38 for details), it was possible to explore the expected relative impact of different frequencies of MDA treatment, both with and without periodic delivery of chemical snail control in both high- and low-prevalence settings. The associated costs of adding snail control were tallied for each strategy, and estimates of the incremental cost-effectiveness of adding mollusciciding to current programs were derived. In both low- and high-prevalence settings, community-wide MDA with additional snail control reduced total disability (measured in disability-adjusted life years [DALYs]) by an additional 40% compared with school-based MDA alone. The optimally cost-effective scenario included the addition of snail control to MDA in more than 95% of simulations. In high-burden settings, annual community-wide MDA with semiannual snail control was, by international health economics standards, highly cost-effective in reducing disease burden, at an approximate cost of US$588 per DALY prevented. These findings support the future inclusion of snail control in global guidelines and national schistosomiasis control strategies, especially in settings with high disease burden or high levels of systematic noncompliance to MDA and in persistent hot spots of transmission.

### Schistosomiasis Collections at the Natural History Museum.

As the SCORE studies were being defined, it became clear that these studies would result in a rich pipeline of schistosomiasis-related materials, and the resultant collections of snails and schistosome genetic material would be a critical resource of long-term value. With assurances from SCORE in regard to providing a consistent pipeline of specimens, a proposal was submitted by the NHM to the Wellcome Trust to support development and maintenance of a specimen repository. The proposal was funded at the NHM, and a repository for storage and research access to schistosome-related specimens, termed SCAN,^37^ was established.

The SCAN project had immediate benefits to SCORE projects because additional staff members were available to undertake fieldwork and provide training for control teams in collecting genetic specimens. The focus on archiving proved beneficial in that SCAN staff logged in specimens, curated and corrected associated data, and stored material in NHM’s purpose-built molecular collections facility, greatly simplifying the work of processing the material while SCORE projects were running. The difficulty of obtaining schistosomes other than highly bottlenecked/inbred laboratory strains has made the SCAN collection very attractive to global research groups working on comparative genomics, with a number of studies in high-profile journals already published using specimens from SCORE and other programs archived in SCAN.^66–68^ Schistosomiasis Collections at the Natural History Museum has also been available to support other projects working in schistosomiasis-endemic regions^16,69^ and to facilitate secondary usage of collections. Although SCAN continues to be a very successful extension of the SCORE program, complications related to implementation of the Nagoya Protocol to the Convention on Biological Diversity may make sharing of genetic samples more challenging in the future, and ways need to be found to facilitate the best research outcomes toward disease elimination at all times, not only during pandemic emergencies.^37^

## LESSONS LEARNED

The SCORE studies on gaining and sustaining control of schistosomiasis^38^ and elimination^49^ add to the growing body of evidence that MDA alone, although often effective in reducing prevalence and intensity of infection, will not result in the elimination of this disease. With increasing interest in achieving elimination in Africa, as demonstrated by the passage of WHA65.21,^6^ and the evidence that prevalence can easily rebound without continued MDA, there is general agreement about the need for integrated approaches to schistosomiasis control and elimination. The rebound of schistosomiasis in high-risk communities can be explained by taking into account the efficiency of human to snail, and snail to human, transmission, and modeling suggests that MDA alone is unlikely to achieve elimination. This view is aligned with the broadest view of control of any infectious disease, incorporating reduction of risk alongside therapeutics. But, in reality, this can be a most challenging and difficult task.

### Monitoring in very low–transmission settings.

Because schistosome larval stages reproduce asexually in the snail host, amplifying the potential impact of even a single infected human on schistosomiasis transmission, focusing on snails is therefore, a necessity. Furthermore, the experience of reducing transmission in countries like Egypt and China, modeling studies, and other evidence indicate that snail control can provide critical additional support for elimination efforts.

Regarding xenomonitoring, it is potentially most critical as human infection rates become low. However, as transmission decreases, xenomonitoring may become more difficult, creating a conundrum when no infected snails can be found, but transmission is ongoing. For example, in the urogenital schistosomiasis outbreak in Corsica, transmission has persisted over several years among people who had not traveled to endemic sites, but no local patent or prepatent snails have been found.^70^ Additional work is needed to find ways to make xenomonitoring more efficient and reduce costs. However, here we have shown that infected snails can be found in low-transmission/prevalence settings, but it is important to identify the species of schistosome involved in the infections.

Monitoring of either intermediate host snail or schistosome DNA in environmental samples offers promise as a complement (or in time an alternative) to traditional monitoring methods, especially in the context of elimination programs when schistosome prevalence or snail abundance may drop to low numbers. This is a field likely to develop rapidly, with many tractable questions awaiting answers, including how long parasite DNA can persist in water, whether eDNA approaches can be sufficiently sensitive and specific to be of value, and whether comprehensive sampling designs can be developed to ensure thorough sampling of suspected transmission sites in ways that are inexpensive, robust, and readily adaptable to local laboratory situations.^71,72^

### Chemical and biological control.

Two SCORE studies explored the impact of using niclosamide to reduce populations of vector snails, *Bulinus* species, to attempt to limit transmission of urogenital schistosomiasis, *S. haematobium*. In both Zanzibar and Côte d’Ivoire, focal snail control using niclosamide was effective in reducing snail populations. However, mollusiciding by itself will not remove 100% of the snails from a water body, and in both studies, snails returned between surveys. The reappearance of snails following chemical control is not surprising because snails can reinvade from untreated areas in large water bodies. Niclosamide is not stable when exposed to sunlight and activity reduces after 24 hours. As *B. globosus* is able to self-fertilize, a population can recover from a single surviving snail. In addition, some species of *Bulinus* can estivate during the dry seasons and, thus, will be untouched by the molluscicide.

Data on human prevalence and intensity from the seasonal transmission study are pending in Côte d’Ivoire. In Zanzibar, the impact of snail control alongside biannual MDA did not make a significant difference in this low-prevalence setting^50^ but did have a great impact on the genetic diversity of the schistosome populations.^41^ Issues related to the study power, study design, and the difficulties in measuring changes in prevalence and intensity when starting prevalence is low are discussed in Ref. 50.

Another important finding in Côte d’Ivoire was the occurrence of *S*. *haematobium–bovis* hybrids in snails, which are known to infect humans and could affect the epidemiology of urogenital schistosomiasis.^41^ The implications of hybrids both in terms of morbidity and in terms of the potential for nonhuman species to serve as a reservoir are not yet clear.

There are several downsides to using niclosamide. In the two SCORE studies that used chemical snail control, the snail surveys and mollusciciding were labor intensive. Many bodies of water were difficult to reach. Also of concern are unintended impacts on other aquatic fauna. As described by Wright,^73^ desirable characteristics of the “ideal” molluscicide include prolonged activity in water at low concentrations, no toxicity to man and warm-blooded animals, harmless to fish and wildlife, non-repellent to snails, cheap, readily available, easily transportable, chemically stable, and agreeable to handle. The experience in the SCORE studies and others shows the need to explore other mollusciding agents, or targeted/baited forms of molluscicides, which have less, or ideally no, impact on nontarget organisms.

The two biological control studies explored the roles of molluscivorous decapods, one an indigenous *Macrobrachium* riverine prawn and the second the introduced crayfish *P. clarkii*, in potentially controlling schistosome-transmitting snails. Although the riverine prawns have many traits that make them potentially good candidates to use in control,^3,55^ at least in the context of rivers and associated habitats in Côte d’Ivoire, there may be insufficient ecological overlap between prawns and schistosome vector snails for prawns to have a sustained and significant impact on transmitting snails.^45^ Conversely, crayfish have several undesirable properties (exotic and invasive, generalist predators, and destructive impacts on earthen dams), but their distribution frequently overlaps with that of pulmonate snails, and their presence is often associated with persistent reduced snail populations in some locations such as Lake Naivasha or Egyptian irrigation canals, illustrating the focal nature and geographic specificity of snail populations. Future evaluations should include assessing their impact on schistosomiasis transmission in the expansive Mwea rice scheme in Kenya and other locations, as it is likely that *P. clarkii* will continue to expand its African range through natural and anthropogenic means. It will be valuable to formally extend evaluation of the impact of *P. clarkii* on native habitats, versus its beneficial role in the control of trematode diseases. As an exotic species, opposition to the use of *P. clarkii* in schistosomiasis control can be anticipated, especially if control efforts involve crayfish introductions beyond their current known African range.

Studies of both prawns and crayfish remind us that a more thorough exploration of the interface between aquaculture, schistosomiasis transmission, and biological control efforts is required. Aquaculture activities, which will be increasingly in demand in tropical Africa,^74^ are likely to directly and indirectly affect schistosome-transmitting snails in ways as yet little studied or appreciated.^75,76^ For instance, we noted at Sirikwa Dam near the town of Eldoret that crayfish were abundant and schistosome-transmitting pulmonate snails were absent in the late 1980s–early 1990s. Following the introduction of catfish in the late 1990s, crayfish numbers declined and pulmonate snails (including potential schistosome vectors) became established. It may be possible, however, to design innovative solutions to take advantage of both crayfish and catfish. By simultaneously introducing fingerling catfish with crayfish into appropriate aquaculture habitats where schistosomiasis is an issue, crayfish may first clear the habitat of schistosome-transmitting snails and may later be consumed by the now-adult catfish. Such a strategy may provide a double bonus in that disease reduction would be accompanied by a much needed new source of protein for local residents. If management problems can be addressed and if biological control agents can be used judiciously—only where they are likely to prove successful without causing environmental harm, they may represent a valuable asset in integrated schistosomiasis control programs.^77^

It is also important to recognize that all future snail control operations will be greatly influenced by the changing environmental circumstances in which we find ourselves. Molluscicide applications are greatly affected by seasonal changes in rainfall and habitat volumes.^49^ The persistence of biocontrol or aquaculture species will be directly influenced by habitat permanence, with crayfish, for instance, being better able to establish thriving habitats in more permanent water bodies such as Lake Naivasha, than in ephemeral pools or other small, man-made impoundments, prone to occasional drying. The ability of some snails to withstand long periods of drying by estivation or dispersal from nearby habitats may enable them to outlast biocontrol agents such as crayfish in ephemeral habitats. If, however, these sites are sufficiently isolated, snails may fail to recolonize them. In such cases, schistosome transmission may remain low, thanks to “The Ghost of Crayfish Past.” Pervasive agrochemical use may unwittingly benefit snails by favoring the algae that snails eat or by killing snail predators such as crayfish.^78^ When schistosome-transmitting snails are considered against the backdrop of rapidly changing environmental conditions, including unanticipated introductions of competitors or predators, rapid alterations in thermal regimes, land-use change, including expanding aquaculture operations, widespread pollution, or habitat drying resulting from irrigation or domestic use, it may well be revealed how fragile the schistosome life cycle can be. All of these changes have the potential to displace or reduce in abundance the obligatory snail hosts of schistosomes.

On top of these strategies, newer gene drive technologies clustered regularly interspaced short palindromic repeats (CRISPR) are being developed within several laboratories. The success of these within other vector-borne disease control programs may provide models that will inform future efforts of the schistosomiasis research community. Challenges specific to schistosomiasis include the hermaphroditic nature of the snails and their genetic and spatial heterogeneity.

### Snail control infrastructure.

Investment in dedicated teams that can identify snails, ascertain level of infection, and conduct treatments must form an integrated part of control programs if elimination is to be achieved. The SCORE’s investment in snail control in Zanzibar and Côte d’Ivoire resulted in enhanced capacity in both those sites. By holding SCORE snail control–focused meetings and through SCORE projects that included snail assessments and control, SCORE brought renewed attention to snails from leadership in schistosomiasis, including at the WHO. In part, because of this renewal of interest, the WHO has held two malacology training sessions in sub-Saharan Africa in hope of bringing back much needed malacological expertise. In addition, the WHO has produced a new manual on snail control for use by present-day NTD programs.^48,79^

## SUMMARY

There is no question that a better understanding of snail biology and the interactions between snails and other organisms, including humans and schistosomes, and the environment will be needed if we are to achieve elimination of schistosomiasis. The SCORE studies have contributed substantially to the body of knowledge about these topics, and have provided data and analyses related to the impact of snail control interventions. In addition, through donations of snail and parasite materials, SCORE has contributed to the establishment of SCAN, which currently assures broad availability of snails and schistosome genetic materials for future investigators seeking to further our understanding and contribute to future schistosomiasis control and elimination efforts.

## Figures and Tables

**Table 2:** March 2014 resurvey of five Kenya schools to understand long-term impact of *Procambarus clarkii* on snails capable of transmitting human schistosomiasis

	Experimental schools			Control schools	
	Kwandoo	Kataluni	Ikotamwithe	Kisukioni	Nzangathi
Number of crayfish captured	0	0	0	0	0
Number of traps placed	18	18	10	18	8
Snails identified					
Number of *B. africanus*	0	0	0	7	8
Number of *B. pfeifferi*	0	0	0	0	0
Number of *L. natalensis*	0	0	0	1	0
Number of *B. forskali*	44	0	41	0	3
Number of *M. tuberculata*	0	0	0	0	0
Number of *P. acuta*	0	0	0	0	0
Number of *Gyraulus* sp.	0	0	1	0	0

In 1989, *P. clarkii* was introduced into aquatic habitats with *Bulinus* snails in three schools, matched with three controls, which did not have crayfish introduced. Human–water contact sites at five of these schools were resurveyed in 2014 for *P. clarkii* and human schistosome–transmitting snails (*Bulinus africanus* and *Biomphalaria pfeifferi*), as well as other species (*Lymnaea natalensis*, *Bulinus forskalii*, *Melanoides tuberculata*, *Physa acuta*, and *Gyraulus* sp.).
